# Commentary: Contextualizing Neuroticism in the Hierarchical Taxonomy of Psychopathology

**DOI:** 10.3389/fpsyt.2020.00175

**Published:** 2020-03-12

**Authors:** Bertus F. Jeronimus

**Affiliations:** ^1^Department of Developmental Psychology, Faculty of Social and Behavioural Sciences, University of Groningen, Groningen, Netherlands; ^2^Interdisciplinary Center Psychopathology and Emotion Regulation, Department of Psychiatry, University Medical Center Groningen, University of Groningen, Groningen, Netherlands

**Keywords:** personality, emotional stability, developmental perspective, longitudinal, happiness, frustration

Although I agree with most of the great review by Brandes and Tackett (1) about the connection between neuroticism and psychopathology as conceptualized in the Hierarchical Taxonomy of Psychopathology (HiTOP), there are several limitations and future research directions on which I wish to elaborate. First, and most fundamental, I welcome the aims of the HiTOP model to overcome some limitations of categorical diagnostic systems (2). However, the HiTOP frequency-approach remains largely similar to the Diagnostic and Statistical Manual of Mental Disorders [DSM-5, (3)] insofar that it negates the time dimension and most intra-individual dynamics and developmental interconnections between the various system levels [e.g., Fisher et al. (4), Jeronimus (5), and Molenaar (6)], from emotions [over hours, e.g., Kuppens et al. (7)] to mood problems [over weeks, e.g., Wichers (8)] and interpersonal and personality processes [over months and years, e.g., Hopwood (9) and Mobbs (10)], their co-development [e.g., Durbin and Hicks (11) and Ormel et al. (12)], underlying processes [e.g., Kunnen et al. (13)] and origins [e.g., Kendler et al. (14); or Nickels et al. (15) for some problems with the HiTOP], among others. For example, in line with the HiTOP model, Brandes and Tackett cover the time dimension only in Table 1 and on page 242. Neuroticism may be linked to the emergence of symptoms and psychopathology [(1), p. 243] but proof requires studies and manipulations that cover *intra*-individual changes across various time scales [e.g., Jeronimus (5) and Hamaker and Wichers (16)]. Hence, the inclusion of developmental and dynamic process-perspectives and methodology would make the HiTOP approach even more relevant and exciting. For example, the 25% of the Dutch population with the highest neuroticism scores seem to generate over 80% of all mental health costs (17), but a strictly dimensional or spectrum perspective cannot explain why many people with high neuroticism scores do *not* develop disorders and are healthy and happy (as illustrated in Figure 1 below)^1^. Health is a multidimensional state and in certain circumstances high levels of neuroticism can benefit health (20), such as when combined high conscientiousness [e.g., healthy neurotic; Turiano et al. (21) and Weston and Jackson (22)] or in interaction with various other personal strengths [e.g., Bos et al. (23) and Tamir et al. (24)]. Furthermore, high scores on the neuroticism facets worry and vulnerability predict longer lives (25). Future work shall show us in more detail what we can learn from healthy and happy neurotics.

**Figure 1:**
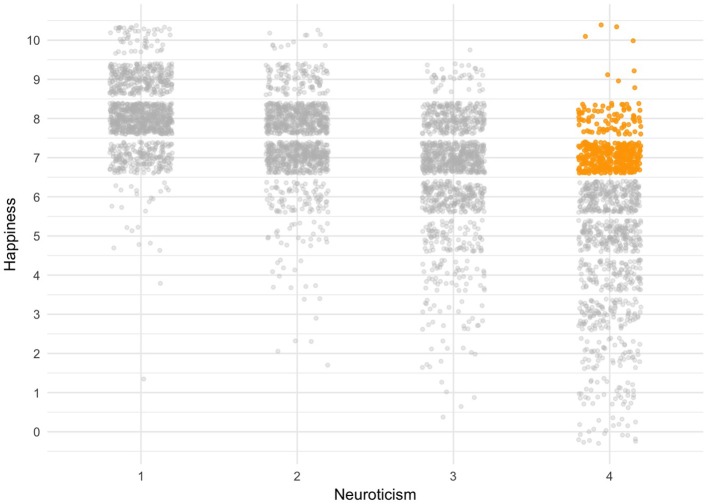
Happy neurotics are depicted in gold. This figure shows 5,000 participants of the HowNutsAreTheDutch study [age range 18–87, mean is 46 (*SD* = 15), 69% women, see van der Krieke et al. (18) for details] who completed the neuroticism scale of the NEO-PI-3 (48 items with Likert scale 1–5). Horizontally you see the neuroticism scale subdivided over four quartiles. The golden section indicates the 10% of the people with the highest neuroticism scores (top 25% or Q4) who report to be happy, which I defined as the top 25% (Q4) of happiness scores [scale 0–10, which ranges from the lowest (=0) to highest (=10) wellbeing one can imagine, see Abdel-Khalek (19)].

Another limitation is the stipulation that neuroticism is the most difficult personality dimension to measure in infants, toddlers, and children [(1), p. 239]. Others have reported that the neuroticism items in self-reported Big Five personality questionnaires may reflect the personality dimension that was most easy to comprehend by children [e.g., Soto et al. (26)]. Moreover, individual differences in negative affect and patterns of emotion dynamics can be reliably *observed* in fetuses (27) and infants (28) and remains the backbone of personality differences along the lifespan [e.g., Houben et al. (29), Jeronimus (30), and Reitsema et al. (31)]. Although we agree that anxiety, sadness, and worry are subjective and potentially hidden within the mind, our avoidance behaviors and inhibitory control, irritability/anger and frustration tolerance surface easily and can be reliably measured [e.g., Caspi et al. (32) and Jeronimus et al. (33)], especially in trait relevant situations [e.g., Hirschmüller et al. (34)]. Next to the question whether the infant or childhood complex of emotions and temperament is “personality” [as the cognitive maturity that is required for most fine-grained and differentiated personality self-descriptions and our narrative identity typically emerges over early adolescence, see De Pauw (26), McAdams (35), and Soto et al. (36)]^2^, it also remains doubtful whether the other broadband factors openness, agreeableness, or conscientiousness/effortful control are more easily and reliably observable in human infants and children [e.g., Goldberg (37) and Mervielde et al. (38)], primates (39), or adults (40, 41) and their environments [e.g., Gosling (42)].

A third limitation is that *frustration* was not mentioned once, despite the strong prospective link between temperamental frustration in children and adolescents and the development of both internalizing (self-directed) and externalizing (other-directed) problems [e.g., Caspi et al. (32) and Jeronimus et al. (43)]. Space constraints may have limited the number of lower-order facets of neuroticism that could be reviewed (see Table 2 on page 239), but frustration is a key feature and temperamental precursor of neuroticism in youth [e.g., Jeronimus et al. (33), Putnam et al. (44), and Rothbart (45)] and adults (46), and if frustration was not part of any of the neuroticism questionnaires that were reviewed, this may indicate a notable limitation in the field of neuroticism assessment. For example, frustration (i.e., unexpected non-reward) *may* lead to irritability (sensitivity/excitability) but could also propel *positive* processes other than anger (displeasure/hostility), anxiety, or sadness [see Jeronimus et al. (33)], which are all prominent states within the neuroticism domain. I wholeheartedly agree with Brandes and Tackett that we must untangle *which* aspects of the multifaceted neuroticism construct predict *what* outcomes in more detail [cf. Hill et al. (25)], via the study of “personality nuances” [e.g., Mõttus et al. (47)] and the inclusion of individual dynamics [e.g., Jayawickreme et al. (48) and Jeronimus and Reitsema (49)], and we have only started to explore such questions. For example, the concurrent and prospective associations between neuroticism and somatic distress [as mentioned by Brandes and Tackett (1), p. 241; see Cuijpers et al. (17), Costa and McCrae (50), and Rosmalen et al. (51)] might primarily reflect vigilance (24) and overlap in semantics and negative affect [e.g., De Gucht et al. (52, 53) and van Diest et al. (54)].

Finally, the review by Brandes and Tackett missed a recent meta-analysis of the prospective associations between neuroticism and psychopathology with 59 longitudinal/prospective studies and 444.313 participants (55). This meta-analysis showed prospective associations between neuroticism and symptoms/diagnosis of anxiety, depression, and non-specific mental distress (*d* = 0.50–0.70) and considerably weaker prospective associations with substance abuse and thought disorders/symptoms (*d* = 0.03–0.20). After adjustment for baseline symptoms and psychiatric history the prospective associations between neuroticism and internalizing phenomena were reduced by half (*d* = 0.10–0.40), whereas the association with substance abuse and thought problems were not attenuated. Prospective associations were four times larger over short (<4 year) than long (≥4 years) follow-up intervals, suggesting a substantial decay of the association with increasing time intervals. Adjusted effects were only slightly larger over short vs. long time intervals, however, which suggests that high neuroticism indexes a risk constellation that exists years prior to the development and onset of all measured mental disorders. Admittedly, such prospective associations do not rule out the spectrum and scar model—see Ormel et al. (56) or Tackett et al. (57) for elaborations, for which novel studies are required [e.g., Bos et al. (58); Goldstein et al. (59)]. Nonetheless, these prospective associations between neuroticism and psychopathology clearly support the integration of emotional, mood, and personality processes across multiple time scales, which may be required to give the HiTOP model mechanistic substance at the individual level and expand our understanding of the dynamic link between neuroticism, psychopathology, and (un)happiness.

## Author Contributions

The author confirms being the sole contributor of this work and has approved it for publication.

### Conflict of Interest

The author declares that the research was conducted in the absence of any commercial or financial relationships that could be construed as a potential conflict of interest.

## Footnotes

^1^Which also requires an explanation if neuroticism is equated to the general factor *P* [see Brandes and Tackett (1), p. 241].

^2^Several aspects of our personality go beyond our inborn temperamental core—via sociocultural influences that are specific to the context in which you acquire your personality—including social cognition and self-related perceptions such as self-esteem and paranoia and social emotions like pride/guilt/shame, and cognitive adaptations to our social world such as the contents of thoughts, attitudes, values, beliefs, morals, needs and goals, as well as the concepts, schema's, and life narratives and identities which subsume tasks, goals, projects, tactics, defenses, values, and other developmental, motivational, and/or strategic concerns that contextualize individual lives in time, place, and role performances. Most of these personality components cannot be distinguished in young children as humans typically develop them over adolescence.
